# Regulation of Selenium/Sulfur Interactions to Enhance Chemopreventive Effects: Lessons to Learn from Brassicaceae

**DOI:** 10.3390/molecules25245846

**Published:** 2020-12-10

**Authors:** Muna Ali Abdalla, Saad Sulieman, Karl H. Mühling

**Affiliations:** Institute of Plant Nutrition and Soil Science, Kiel University, Hermann-Rodewald-Str. 2, 24118 Kiel, Germany; ssulieman@plantnutrition.uni-kiel.de

**Keywords:** selenium, sulfur, acquisition, assimilation, biofortification, hyperaccumulator, Brassicaceae, Se/S ratio, chemopreventive effects

## Abstract

Selenium (Se) is an essential trace element, which represents an integral part of glutathione peroxidase and other selenoproteins involved in the protection of cells against oxidative damage. Selenomethionine (SeMet), selenocysteine (SeCys), and methylselenocysteine (MeSeCys) are the forms of Se that occur in living systems. Se-containing compounds have been found to reduce carcinogenesis of animal models, and dietary supplemental Se might decrease cancer risk. Se is mainly taken up by plant roots in the form of selenate via high-affinity sulfate transporters. Consequently, owing to the chemical similarity between Se and sulfur (S), the availability of S plays a key role in Se accumulation owing to competition effects in absorption, translocation, and assimilation. Moreover, naturally occurring S-containing compounds have proven to exhibit anticancer potential, in addition to other bioactivities. Therefore, it is important to understand the interaction between Se and S, which depends on Se/S ratio in the plant or/and in the growth medium. Brassicaceae (also known as cabbage or mustard family) is an important family of flowering plants that are grown worldwide and have a vital role in agriculture and populations’ health. In this review we discuss the distribution and further interactions between S and Se in Brassicaceae and provide several examples of Se or Se/S biofortifications’ experiments in brassica vegetables that induced the chemopreventive effects of these crops by enhancing the production of Se- or/and S-containing natural compounds. Extensive further research is required to understand Se/S uptake, translocation, and assimilation and to investigate their potential role in producing anticancer drugs.

## 1. Introduction

Cancer is a big challenge globally and one of the leading causes of death. According to the World Health Organization, cancer is responsible for one in six deaths, which makes it the second most common cause of death worldwide: 9.6 million deaths from cancer were reported in 2018 [1]. Therefore, there is an urgent need for the development of new antitumor agents with high efficiency and low toxicity. In this regard, a promising example could be natural products from plants and food.

Previous epidemiological investigations have demonstrated a relationship between selenium concentrations in crops and mortality rates [2,3]. Additionally, case-control and promising studies have shown that people with low serum selenium levels have an elevated risk of developing cancer [4,5].

Metabolites containing sulfur-based chemical linkages such as disulfide, thioether, and thioketal bonds have been widely used for the development of tumor-specific, redox-responsive drug delivery systems (DDSs) [6,7,8,9]. Besides the redox responsivity stimulated by S bonds, Se, which is in the same group as S in the periodic table, has gained significant attention for its redox responsivity. Se is an essential trace element for plant, humans, and animals that plays a significant role in antioxidant defense and controls the cell redox status [10]. A series of selenoether- or diselenide-containing polymers have been designed to establish redox-responsive DDSs for cancer treatment [11,12].

Both Se and S share common metabolic pathways in plants owing to their chemical and physical resemblance and, interestingly, Se can act as a substitute for S in physiological and metabolic processes [13,14]. For example, it is incorporated into Se-amino acids, which can replace S-amino acids in the formation of the proteins [15].

Moreover, Se and S isolog compounds show that these elements interact with each other and compete in biochemical processes; therefore, their uptake, translocation, and assimilation at different times of plant development can be affected depending on the Se/S ratio. Plants are considered as an important dietary source of Se for animals and humans. Two forms of Se—selenate and selenite—are available for plants, depending on the soil chemical characteristics.

In a previous study of the accumulation and volatilization of Se in Indian mustard (*Brassica juncea*), it was found that selenate was taken up two-fold faster than selenite [16]. Therefore, selenate is known to be the most common source of Se in soils, which is transported inside the plant through sulfate transporters [17]. Subsequently, Se can be translocated and metabolized in plastids and assimilated through the S assimilation pathway into Se-amino acids [18].

SeCys exists in at least 25 various selenoproteins, such as several thioredoxin reductases in addition to glutathione peroxidases. Thus, SeCys has been responsible for the health benefits of Se [19]. As a result, Se is classified as an essential microelement for humans and animals. Se intake in the human diet is often lower than the daily recommended dose of 50–70 μg, which is needed for maximal expression of selenoproteins [20]. Therefore, Se deficiency is a global issue, which is linked with several diseases including hypothyroidism, male infertility, cognitive decline, cardiovascular disease, weak immune system, and rising prevalence of different cancers [21,22,23,24].

In this review, we highlight the influence of Se and Se/S nutrition in Brassicaceae and their potential benefits to human health including their chemopreventive properties. Se and S acquisition, translocation, and assimilation in relation to their accumulation were discussed and several experiments in brassica vegetables were documented. Such approaches might assist the development of nutraceutical functional foods with high chemoprevention efficacy. 

## 2. The Distribution of Selenium and Sulfur Compounds in Brassicaceae and Their Chemopreventive Potency

Brassicaceae vegetables are one of the dominant food crops globally. For example, *Brassica oleracea* includes the important staple food cultivars such as cabbage, broccoli, brussels sprouts, kohlrabi, kale, and cauliflower [25]. They are recognized for their characteristic flavor and functional properties, which are directly associated with the unique natural compounds that they contain [26]. Brassicaceae are well known for their high sulfur levels and interesting S-containing metabolites such as glucosinolates (GLS) and indole-type phytoalexins [27,28]. The hydrolyzation of the GLS by the myrosinase enzymes that are found in brassica vegetables results in the production of a variety of biologically active compounds including isothiocyanates (ITCs), thiocyanates, nitriles, and epithionitriles. Importantly, ITCs are responsible for both the pungent aromas as well as the potential bioactivities [29] such as anticarcinogenic [30,31], antimicrobial, and anti-inflammatory effects [32]. Moreover, ITCs are linked to the anticarcinogenic effects of brassica vegetables owing to their multiple cancer-preventive bioactivities, including chemopreventive phase-I enzyme inhibition and phase-II detoxification enzyme induction, in addition to its potential role in the induction of apoptosis as well as cell cycle arrest [32]. The most comprehensively investigated anticancer isothiocyanate is sulforaphane, which is isolated from cruciferous vegetables, mainly from broccoli and broccoli sprouts [33]. Sulforaphane has shown remarkable effects towards lung, prostate, breast, ovarian, and melanoma cancers [34]. Erucin, a known antitumor agent, is derived from glucosinolate glucoerucin by the action of the myrosinase enzyme when the vegetables are chewed or chopped. High quantities of glucoerucin are accumulated in brassica crops including many varieties of Chinese cabbage and kohlrabi, in addition to arugula and wild rocket (*Eruca sativa* (Mill.) [35,36].

The phytoalexins brassinin 1-methoxybrassinin and cyclobrassinin isolated from the Chinese cabbage heads of *Brassica campestris* inoculated with the bacterium *Pseudomonas cichorii* and spirobrassinin obtained from the roots of canola (*Brassica napus*) infected with phytopathogenic *Plasmodiophora brassicae* (clubroot) have demonstrated a cytotoxic effect [37,38,39]. Additionally, 1-methoxyspirobrassinol and 1-methoxyspirobrassinol methyl ether obtained from the Japanese radish “Daikon” after inoculation with *Pseudomonas cichorii* [40] showed antiproliferative activity [41]. The anticarcinogenic properties of Se-containing compounds, including SeMet, methylselenol (CH_3_SeH), and MeSeCys, have been reported [42,43,44,45]. El-Bayoumy and Sinha (2004) indicated that MetSeCys reduces 7,12-dimethylbenz(a)anthracene (DMBA)-induced mammary tumors and stops cancer progression and proliferation of precancerous mammary lesions [46]. It has been previously suggested that selenobetaine and MeSeCys were more promising chemopreventive compounds in comparison with Na_2_SeO_3_ and SeMet, as investigated in the carcinogen-induced rat mammary carcinogenesis model. Therefore, this has gained increasing attention as reported in several studies, which have indicated that methylseleninic acid (CH_3_SeO_2_H), known as a direct precursor of CH_3_SeH, reacts more quickly than MeSeCys in suppressing the synthesis of DNA in in vitro mammary cancer cell lines [47,48,49]. Additionally, CH_3_SeH, was found to afford selenocompounds that induce the efficiency of mammaglobin-A peptide vaccination toward breast cancer cells [50]. MeSeCys and γ-glutamyl-Se-methylselenocysteine (γGluMeSeCys) are among the important monomethylated forms of Se, which are precursors of the potent chemopreventive compound CH_3_SeH [51]. MetSeCys is a plant-derived, naturally occurring compound, which is found in high levels in brassica plants, that have the ability to accumulate Se [52]. It has been discovered that some Brassica and Allium species are able to accommodate 0.1–2.8 μmol g^−1^ DW (dry weight) MeSeCys or its functional equivalent γ-GluMeSeCys [53,54].

**Figure 1:**
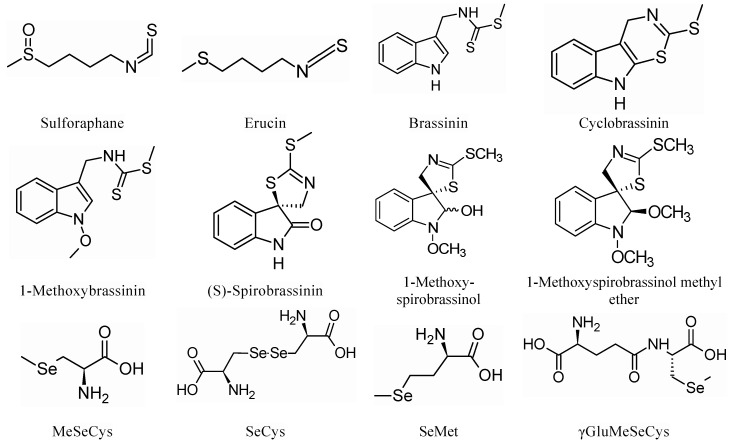
Some naturally occurring organosulfur and organoselenium compounds obtained from Brassicaceae with chemopreventive effects.

Some naturally occurring organosulfur and organoselenium compounds obtained from Brassicaceae with chemopreventive effects are listed in Figure 1.

It is important to mention that the mechanisms by which Se might reduce oncogenesis or act as a chemopreventive agent have been reviewed by many authors [43,55]. It has been reported that the most possible mechanism of Se as an anticarcinogenic is its role as a modulator of antioxidant defense systems to decrease oxidative stress and protect cells from DNA damage. In this regard, the thioredoxin-thioredoxin reductase system is over-expressed in human cancer; therefore, the important selenocysteine-containing protein exhibited protection against oxidative stress and anti-apoptotic functions. Consequently, Se-containing antioxidant system regulates the activity of different enzymes and maintains redox status in cells [56]. Contrary to the antioxidant potential of Se, the second Se anticarcinogenic mechanism reveals Se’s opposite role as pro-oxidant [55,57,58,59]. Selenocompounds such as selenol derivatives can react with O_2_ to produce superoxide and other reactive oxygen species (ROS). For instance, the addition of methioninase and β-lyase enzymes to SeMet and MeSeCys, respectively, directly generates significant amounts of CH_3_SeH, which might be oxidized to methylseleninic acid or react with O_2_ to produce superoxide and ROS.

Selenite is also known as a particularly good oxidant. It can be reduced by glutathione (GSH) to obtain glutathione selenopersulfide (GS–SeH), selenodiglutathione (GS–Se–SG), and other species that can produce CH_3_Se−, which is able to generate ROS when higher selenite supplementation is consumed [57,60]. ROS exhibit a central role in triggering apoptosis. Consequently, tumor cells that are primed for apoptosis thus induce the suicide of precancerous and cancerous cells while leaving nonmalignant cells untouched.

It has been reported that Se biofortification might modify the synthesis of phenolic acids and amino acids [61], which are found in cruciferous vegetables. Phenolics and amino acids are known as N-containing compounds; therefore, N metabolism is significantly affected by Se, owing to its interaction with the biosynthetic pathway of S-compounds, and/or decreases molybdenum acquisition as an essential cofactor for the activity of nitrate reductase, which is important for the utilization of nitrate by plants [62].

## 3. Selenium-to-Sulfur Ratios and Their Accumulation in Brassica Vegetables

Owing to the close chemical similarities between sulfur and selenium, Se often substitutes for S in metabolic and physiological processes depending on the growth conditions including the Se/S ratio in the growth medium and/or in the plant [63].

Brassicaceae along with a number of members related to Asteraceae and Fabaceae are known as Se-accumulator species owing to their ability to accumulate Se concentrations, which exceed 1 mg Se g^−1^ DW in leaf trichomes and epidermal cells [64].

It has been reported that Se predominantly improves plant growth and stress tolerance at 1–10 mg Se/kg DW [65]. Additionally, Se toxicity is determined when the Se tissue concentration reaches >100 mg Se/kg DW [66,67]. However, the capability of plants to accommodate Se significantly differs; therefore, they are classified into three categories: nonaccumulators, secondary accumulators, and hyperaccumulators. Nonaccumulators have low levels of Se, which can be less than 100 µg Se g^−1^ DW. Secondary accumulators are able to grow on soils with Se content ranging from low to medium and can accommodate up to 1000 µg Se kg^−1^ DW; good examples are *B. juncea* and *B. napus*. Hyperaccumulators thrive on seleniferous soils with 2–10 ppm Se and have the capacity to concentrate Se at more than 1000 µg Se g^−1^ DW, including several species of the genera *Astragalus* (Fabaceae) and *Stanleya* (Brassicaceae) [68]. White et al. (2004) studied the interaction between Se and S in the nonaccumulator *Arabidopsis thaliana* (L.) Heynh. plants grown on agar plates and treated with varied concentrations of S and Se [69]. The authors found that the Se/S concentration ratio in the shoot was dependent on the selenate/sulfate concentration ratio in an agar medium. In this regard, an elevated S concentration in agar enhanced the S level and shoot fresh weight and reduced the shoot Se concentration. On the other hand, a higher Se concentration in agar increased both the Se and S levels in shoot but decreased the shoot fresh weight. Additionally, a decreased shoot fresh weight under Se treatment was linearly associated with the shoot Se/S ratio. The authors suggested new evidence for a physiological role of various sulfate transporters in *Arabidopsis* roots with different sulfate/selenate selectivities that control transport through the plasma membrane in response to nutritional conditions.

Galeas et al. (2007) investigated Se/S seasonal fluctuations in hyperaccumulator plants *Stanleya. pinnata* (Pursh) Britton and *Astragalus bisulcatus* (Hook.) Gray in addition to nonaccumulators including *Astragalus sericoleucus* Gray, *Oxytropis sericea* Torrey and A. Gray, and *Thlaspi montanum* L. The authors indicated that the hyperaccumulator plants demonstrated decreasing pattern in leaf Se concentration from spring to autumn. Additionally, the Se level had a specific flow in hyperaccumulator plants, in which the lowest root Se concentration was detected in summer. The authors noticed that Se showed flow from the root to young leaves and subsequent Se remobilization was detected from old leaves to reproductive parts in summer, in addition to subsequent release in roots in the autumn season. In contrast, the nonhyperaccumulators had the highest Se concentrations in summer. S had the highest level in summer in both hyperaccumulator and nonhyperaccumulator species [70]. Regarding Se/S interaction, the authors mentioned that comparing soil with plant materials’ concentrations did not show significant correlations for Se or S, which confirmed that their acquisition and localization significantly regulated the process. For example, a 50-fold increase in the Se/S ratio in the leaves compared with that in the soil suggested that the studied hyperaccumulator plants took up Se in preference to S. Owing to the presence of one or more sulfate transporters in the hyperaccumulator plants, which might be developed into Se-specific transporters, this can explain the 100-fold increase in the Se/S ratio in these plants. On the other hand, 100-fold lower Se/S ratios have been observed in nonhyperaccumulators. It is not stated that sulfate transporters in nonhyperaccumulators take up S preferentially over Se. Consequently, Se and S acquisition can be affected equally, which demonstrated the significant correlation between Se and S tissue accumulations. These findings clearly agreed with the results reported by El Mehdawi et al. (2018), who studied the impact of sulfate supply on Se acquisition dynamics in addition to the expression of sulfate/selenate transporters in Se hyperaccumulator and nonhyperaccumulator brassica plants. The authors found that *S. pinnata* exhibited a three-fold increased short-term (1 h) Se acquisition rate when determined in the absence of S. Moreover, when S was present, *S. pinnata* specified Se more that nonaccumulators and was able to accommodate higher Se in the presence of a 100-fold higher competing S level. The authors suggested that the hyperaccumulator plants have higher Se over S transport specificity and less repression of Se/S transporters by high S levels [71].

A previous report compared the sulfur-dependent selenite uptake and translocation between the hyperaccumulating plant *S. pinnata* and the nonhyperaccumulating plant *Brassica juncea*. In addition, the authors studied different expression levels of three sulfate/selenate transporters (Sultr) as well as three ATP sulphurylases (APS) in both species. The authors found that Se accumulation reduced ~10 fold in response to a higher sulfate level in *B. juncea*, which was in agreement with the findings of White et al. [69] in *Arabidopsis thaliana*, whereas a 2–3 fold difference in Se uptake was detected in *S. pinnata* containing an increased (5 mM) and decreased sulfate (0 mM) level. Moreover, the hyperaccumulating plant *S. pinnata* had a higher Se/S ratio than the nonhyperaccumulating plant *B. juncea* [72]. Additionally, in Se hyperaccumulators, Se treatment minimized S accumulation in tissues as both S and Se compete for the same transporters, but in these plants the sulfate transporters as well as the enzymes including APS genes are consistently upregulated, which resulted in no S deficiency response. In a recent study in pak choi *Brassica rapa* L. var. Chinensis, which is classified as a secondary Se-accumulating plant, a Se concentration up to 442 μg Se/g DW did not show any signs of toxicity [14]. 

The impact of Se doses on broccoli (*B. oleracea* L. var. *italica*) growth under sulfur-deficient and -sufficient conditions has been investigated. The authors found that *plants* can suffer *Se toxicity* in response to S starvation. The application of Se treatments without S nutrition significantly diminished plant sizes. In contrast, Se toxicity can be highly reduced under appropriate S treatment. Moreover, the authors observed elevated Se toxicity with selenate compared with selenite as a response to poor S supply. Additionally, the authors discovered that high Se toxicity under S deprivation was precisely incorporated with elevated Se in proteins versus total Se concentration. Subsequently, the generation of reactive oxygen species was induced in addition to lipid peroxidation, resulting in cell membrane deterioration and reduced antioxidant enzymes’ potential. The authors suggested that Se toxicity could be curbed by sufficiently enhancing the S supply, which would increase the inhibitory effect of nonspecific integration of Se into proteins in addition to *modulating* the *activities* of the *redox* *enzyme* (i.e., *ascorbate peroxidase and catalase*) [73]. Importantly, the data provided by Tian et al. [73] shed light on Se/S interactions and demonstrated excellent evidence of the importance of S nutrition to avoid Se toxicity when a Se dosage is applied to crops. In this regard adequate S fertilization will enhance beneficial effects of Se biofortification.

**Figure 2:**
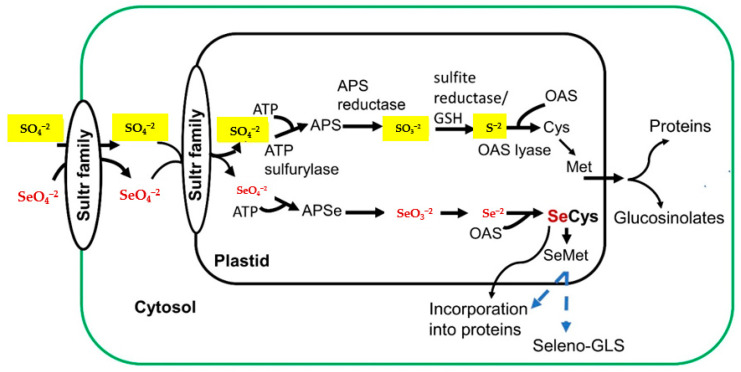
Sulfate and selenate acquisition and assimilation pathways in Se accumulators [74]; Sultr; sulfate/selenate transporters; ATP sulfurylase; adenosine-5’-triphosphate sulfurylase, APS; adenosine-5’-phosphosulfate, OAS; *O*-acetylserine, APSe; adenosine phosphoselenate. GLS; glucosinolates.

The assimilation of Se into organoselenium compounds that behave very similarly to their organosulfur analogues is thought to be a result of competition with S assimilation. Therefore, SeCys and SeMet can get nonspecifically incorporated into proteins (Figure 2), whereas misincorporation might be the main reason for Se toxicity in Se nonaccumulating plants. Consequently, in the hyperaccumulator plants the synthesis of SeMet might be limited and the SeCys is transformed to nonprotein seleno amino acids including MeSeCys, selenocystathionine, and the potent chemopreventive selenium peptide γ-glutamyl-MeSeCys [69].

A previous study detected putative selenoproteins in Chinese cabbage (*Brassica pekinensis* L.) cultivated in field trials under Se treatments. The authors indicated that several putative selenoproteins with a greater range of molecular weights from 10 to over 100 kD were observed. Moreover, the authors reported that the presence of considerable proportions of Se protein suggested the incorporation of Se into specific proteins rather than being nonspecifically and randomly distributed throughout the biosynthesized proteins. [75].

## 4. Reported Studies about the Impact of Se or Se/S Biofortification on Chemopreventive Inhibitory Effects in Brassica Vegetables

Owing to the potential benefits of Se and its positive impact on human and animals’ health, significant attention has been paid to increase its concentration in foods [76]. However, Se toxicity has been reported in people few days after ingesting of high doses of a dietary supplement [77].

Se and S nutrition are uniquely recognized for their important role in regulation of GLS metabolism, especially in Brassiaceae [78].

The influence of Se as well as Se/S fertilization on the quality and secondary metabolites’ production as well as health benefits including chemopreventive properties of edible brassica crops has been investigated in several studies. For instance, Sams and coworkers studied the effect of elevated Se and S levels on GLS and ITCs in *Arabidopsis thaliana* and a rapid-cycling *B. oleracea*. The authors used HPLC with a photodiode array detector for the determination of GLS, whereas ITCs were identified by means of gas chromatograph using a flame ionization detector (GC-FID) and GC-MS. The authors found that S and Se concentrations were enhanced in both *A. thaliana* and *B. oleracea* under elevated Se doses. Additionally, aliphatic and total GLS reduced significantly (*p* ≤ 0.001) from 0.0 to 3.2 mg Se L^−1^ in both *B. oleracea* and *A. thaliana* leaf tissues. The authors found that S treatment did not affect GLSs’ and ITCs’ levels. Glucoerucin was the only GLS that had a significantly increased concentration, by 28.6% with higher dose of S. These findings are promising for obtaining adequate Se and GLS concentrations in the diet [79].

Additionally, a previous report studied the antiproliferative activity of broccoli biofortified with Se in three different maturity stages toward five human cancer cell lines including glioma (U251), breast (MCF-7), kidney (786-0), lung (NCI-H460), and colon adenocarcinoma (HT-29) [80]. The authors found that the seedlings of broccoli in comparison with sprouts and inflorescences demonstrated the highest phenolic compounds’ accumulation and antioxidant potential. The authors indicated the antiproliferative effect of various broccoli maturity stages biofortified with Se (Table 1), especially seedlings, and recommended them as a promising source of anticancer agents.

Dall’Acqua et al. (2019) reported the impact of selenate fertilization at 0–40 µM on two rocket species, salad and wild rockets, on the profile and content of S-containing compounds and other phytochemicals [81]. The authors found that Se biofortification at or more than 10 µM has a distinct influence in both species regarding the transcription of genes involved in S assimilation and S-containing compound accumulation. The authors suggested that the differences in Se and SeCys accumulation between *E. sativa* and *D. tenuifolia* could explain the diverse regulatory mechanisms of Se/S acquisition and assimilation owing to Se application, which might be associated with varied S concentrations, especially the high S level in *D. tenuifolia*, in addition to the functional properties of assimilatory enzymes and sulfate transporters, known as SULTR.

Moreover, it has been indicated that the concentration of the total GLS in rapid-cycling *B. oleracea* reduced from 5.84 µmol g^−1^ to 1.90 µmol g^−1,^ owing to the influence of sodium selenate (Na_2_SeO_4_) fertilization. Additionally, the levels of 4-methylsulphinylbutyl glucosinolate decreased by 90% in response to an increased Na_2_SeO_4_ supply from 0 to 1 mg L^−1^, whereas it remained low under a higher selenium concentration. Additionally, the authors found that sulfate uptake was enhanced in *B. oleracea* with elevated Na_2_SeO_4_ concentration from 0 to 9 mgL^−1^ [82].

Freeman et al. (2006) studied the spatial Se distribution and quantification of Se compounds in the hyperaccumulators *A. bisulcatus* and *S. pinnata* biofortified with Se using micro scanning X-ray fluorescence mapping (m-SXRF) and liquid chromatography-mass spectrometry (LC-MS). The authors found that Se was accommodated in trichomes in the young leaves of *A. bisulcatus*. Additionally, micro X-ray absorption spectroscopy in addition to LC-MS revealed that Se compounds in trichomes were MeSeCys (53%) and γ-glutamyl-MeSeCys (47%) [83].

Moreover, both MeSeCys (88%) and selenocystathionine (12%) were detected inside the young leaf edges of *S. pinnata*.

The influence of Se and S fertilization on cabbage plants has been evaluated. Additionally, the authors studied the biosynthesis of GLS such as the aliphatic GLS glucoraphanin, known as the precursor of the anticancer metabolite sulforaphane [84]. The authors indicated that the synthesis of GLS was enhanced by exogenous selenite-elevated sulfate supply with the combination of 50 μΜ selenite + 1 mΜ sulfate or 100 μΜ selenite + 4 mΜ sulfate [84]. Previous reports indicated that the resulting high Se concentration might replace S-containing amino acids with Se-containing amino acids and induce S-starvation responses via feedback inhibition of gene expression, which can be alleviated even if S is present. Consequently, in response to S-deficiency and even if moderate concentration of Se occurs, the plant can accommodate high levels of selenium [85,86].

The influence of Se on both green cabbage and red cabbage has been investigated. In this study, Se soluble compounds were extracted from cabbage using protease XIV and identified by ion exchange high-performance liquid chromatography inductively coupled plasma-mass spectrometry (HPLC-ICP-MS). The authors found that 49%, 59%, and 65% of soluble Se compounds were from roots, leaves, and stems, respectively, whereas in red cabbage 28%, 31%, and 43% of soluble Se were detected in roots, stems, and leaves, respectively [87]. The insoluble Se was 31–53% and the authors suggested these amounts might be a part of a detoxification mechanism.

Ávila et al. (2014) studied Se biofortification of different cultivars of six globally consumed cruciferous crops including green cabbage, Chinese cabbage, broccoli, kale, cauliflower, and Brussels sprouts. The authors found that different types and concentrations of GLS were detected in each brassica vegetable. For instance, broccoli sprouts had high levels of glucoraphanin, whereas cauliflower sprouts demonstrated high accumulation of the total GLS [52]. 

The anticancer effects of broccoli sprouts owing to their bioactive primary compound, sulforaphane, has been reported; they showed inhibitory effects against skin and urinary bladder carcinogenesis in vivo [88,89]. Additionally, they reduced human bladder and prostate cancer cells’ proliferation in vitro [90,91]. Abdulah et al. (2009) evaluated the anticancer potential against human prostate cancer cell lines of broccoli sprouts biofortified with selenium [92]. The authors found that selenium-enriched broccoli sprouts’ extract accommodate Se in the form of MeSeCys. These results were in agreement with a previous report, in which the major constituent in broccoli sprout seeds grown in an inorganic Se solution was MeSeCys, which subsequently assimilated into various Se analogs of different S compounds via the S metabolism pathway [93]. The authors discovered that Se-enriched broccoli sprouts reduced proliferation of cancer cells using different mechanisms. Se enrichment might induce the inhibitory effects of cancer cell proliferation and enhance apoptosis of control broccoli sprouts. Additionally, Se minimizes the risk of cancer duplication after release from control broccoli sprouts’ treatment [92].

In a previous study, Matich et al. demonstrated that treatment of broccoli and cauliflower florets, in addition to roots of forage rape, with sodium selenate resulted in the formation of selenoglucosinolates bearing a methyl seleno alkyl moiety [94]. The pentane/ether extracts were analyzed by means of GC-MS and six organoselenium metabolites, including the selenium analogues of myrosinase-derived brassica volatile compounds, were detected: 5-(methylseleno)pentanenitrile, 4-(methylseleno)butanenitrile, 3-(methylseleno)propylisothiocyanate, 4-(methylseleno)butylisothiocyanate, and 5-(methylseleno)pentylisothiocyanate. Additionally, the ethanolic extracts were analyzed using LC-MS to afford three selenoglucosinolates. The authors used LC-MS/MS to define the selenium atom’s position in the selenoglucosinolate and elucidated the amalgamation of selenium atom through SeMet into the methylselenyl group instead of sulfate or β-thioglucose moieties. The authors suggested that the incorporation of elevated concentration of selenium into brassica GLS exhibited a potential strategy to produce selenoglucosinolates in these species, which is reported to have high levels of organosulphides. Therefore, Se supplementation to brassica vegetables containingGLS might provide remarkable health benefits [94]. It is important to mention that selenoglucosinolates afforded by Se biofortification have health benefits: The resulting Se-containing ITCs demonstrated higher anticancer potential than S analogues [95].

Palomo-Siguero et al. (2015) studied the bioefficiency of Se fertilization in radish plants with chitosan-modified Se nanoparticles (CS–SeNPs) using a hydroponic system. HPLC was performed in addition to asymmetrical flow field flow fractionation (AFFFF) and ICP-MS, in conjunction with transmission electron microscopy (TEM) [96]. The authors found that Se accumulation was 25% lower in the case of the application of CS–SeNPs in comparison with selenite treatment. It is important to state that CS–SeNPs are the initiative approach of Se being efficiently biotransformed from NPs into plant species (Table 1).

Se biofortification in kale plants using sodium selenite or sodium selenate has been evaluated. The authors found that kale plants were able to accommodate over 20 times more Se in the selenate-enriched soil in comparison with selenite-enriched soil. The authors combined size-exclusion chromatography and ion-pairing reversed-phase chromatography with (ICP-MS) in addition to nano-electrospray ion trap mass spectrometry (nanoESI-ITMS) to identify Se speciation. The authors indicated that MeSeCys was present in the stem at elevated levels compared with that in the leaf, whereas higher amounts of Se species with high molecular weight were found in the leaf compared with the stem. The kale plant leaf fertilized with Se (IV) produced the highest level of Se metabolites of high molecular weight (49%) in addition to the lowest content of MeSeCys (4%) (Table 1) [97]. The combination of ICP-MS and ESI-MS is a powerful tactic, which can be used to investigate Se metabolites in brassica vegetables. 

Peñas et al. (2012) investigated the effect of selenite in sauerkraut on Se biotransformation, indole glucosinolate breakdown products, vitamin C, and microbial quality in addition to the sauerkraut antioxidant potential and its inhibition of lipopolysaccharide-induced nitric oxide production.

The authors found that the total Se concentration was up to 1.29 μg/g DW. Additionally, in response to Se supplementation, a minor decrease in the level of ascorbigen (6%) in addition to vitamin C (5%) in sauerkraut (*p* ≤ 0.05) was observed. Nevertheless, enhanced lactic acid bacteria (3%) was detected, in addition to the remarkable accumulation of indole-3-carbinol and indole-3-acetonitrile, which has an indirect defensive benefit [98]. Se-enriched cabbage extracts exhibited a promising antioxidant effect (163 μmol trolox/g DW) and suppression of NO production in LPS-induced macrophages (IC_50_ = 44.0 μg/mL) in comparison with the nontreated cabbage extracts (control) [99].

Ouerdane et al. (2013) studied Se species present in black mustard cultivated in Se-rich soil. The authors performed Se-specific detection by means of electrospray ionization (ESI) tandem MS in combination ICP-MS. Additionally, size-exclusion LC-ICP MS was used to identify Se species based on the molecular weight in addition to monitoring their stability during extraction. Over 30 seleno compounds were detected along with minor metabolites (Table 1). Moreover, methylselenonitriles and methylselenoisothiocyanates were characterized using GC-ICP MS coupled with atmospheric pressure chemical ionization (APCI) MS/MS. Although selenocompounds were identified using the developed techniques, the authors did not determine the biological activities of the Se species, which may exhibit remarkable anti-inflammatory and antioxidant effects in addition to cancer prevention [100].

The effect of Se supplementation on kale seedlings has been investigated. The authors used ion- pairing, reverse-phase HPLC–ICP-MS to identify Se species. The total Se content of biofortified kale plants increased in comparison with the control. Kale was able to accumulate 400 μg g^−1^ Se DW when the plants were supplemented with 30 μg mL^−1^ Se. Importantly, the authors noticed that harvesting after more than 15 days afforded approximately 100% of Se in the extract as nonmetabolized selenite [101].

The impact of Se biofortification on the production of cancer chemopreventive compounds in broccoli sprouts and florets has been studied. Selenate and selenite had the same effect in boosting MeSeCys production in sprouts. The authors discovered that the five studied broccoli cultivars showed that, in contrast with florets, sprouts were able to transform inorganic Se to MeSeCys. Additionally, both sprouts and florets afforded an amazing GLS profile, whereby sprouts were found to synthesize the chemopreventive compound glucoraphanin 6-fold more than florets (Table 1). Additionally, the GLS level in florets was not influenced by Se fertilization in sprouts. The study of Ávila et al. (2013) demonstrated that Se-enriched broccoli sprouts are a great approach for the induction of anticancer metabolites including MeSeCys and glucoraphanin [102].

Bañuelos et al. (2015) determined the total Se content and various Se species in broccoli planted in Se-amended soil, which contained ground shoots of the Se hyperaccumulator *S. pinnata*. The authors found that the total Se level enhanced to nutritionally typical levels with increasing treatments of *S.* *pinnata*. The authors used real-time strong anion exchange-HPLC followed by ICP–MS detection (SAX-HPLC-ICP-MS) to detect Se metabolites in aqueous extracts [103]. The authors identified selenocompounds, as listed in Table 1. The addition of Se-enriched *S. pinnata* shoots to the soil to obtain Se-biofortified broccoli was an amazing approach, which showed the bifunctionality of *S. pinnata* as a potential Se phytoremediation tactic and an efficient source of organic Se fertilizer for induction of chemopreventive compounds.

Bañuelos et al. (2016) evaluated the growing of broccoli plants in previously Se-enriched soil that amended with hyperaccumulator plant *S. pinnata* three years before sowing the plants. The authors found that after each particular planting season, the total Se level in broccoli florets ranged from 6.99 to 7.83 mg kg^−1^. Se speciation was determined in aqueous extracts at the elevated amendment rate. A previous report by Bañuelos et al. [103] indicated that Se speciation did not remarkably differ in broccoli cultivating in soils amended with varied amounts of *S. pinnata*. Bañuelos et al. (2016) indicated that SeMet was the prevailing selenoamino acid found in Se aqueous extracts; the other Se species are listed in Table 1 [104].

The use of Se-amended soil to produce broccoli plants containing selenium chemopreventive compounds is an example of a potential organic fertilizer, which can be applied in Se-starved areas as a promising source of Se. 

Kahakachchi et al. (2004) investigated Se biofortification using selenate and selenite in the Se-accumulating plant Indian mustard and identified organoselenium compounds by HPLC-ICPMS. Se species profiles were compared with Se-enriched yeast using HPLC-ICP-MS and GC coupled with atomic emission detectors (GC-AED).

The authors found that the major Se species in the samples supplemented with selenate was inorganic Se (Na_2_SeO_4_), which constituted 82.8% of the eluted Se. The metabolites were characterized as MeSeCys, SeMet, and S-(methylseleno)cysteine. On the other hand, plants treated with Na_2_SeO_3_ afforded SeMet Se oxide hydrate (51.2%) and SeMet (34.2%), which were detected as the major Se compounds. Moreover, selenite was found at a low concentration (4.3%). The authors suggested that the observed elevated concentration of SeMet Se-oxide hydrate, which was not delivered in plants biofortified with selenate, might suggest various redox reactions occurring in these two different treatments [105].

## 5. Conclusions and Future Perspectives

An estimated 1 billion people globally are at risk of Se deficiency, possibly because of insufficient Se intake. Se is required to prevent cardiovascular disease, cancer, virus infections, infertility, and neurological disturbances [106]. Brassica species are sulfur-rich vegetables, which are associated with various health-promoting effects, such as suppression of cancer incidence and its progression [107]. Therefore, there is growing evidence of the cancer chemopreventive and further health benefits of both Se and S biofortification as an effective and potential strategy.

Most of the reviewed studies showed that the supplementation of inorganic Se under hydroponic conditions can deliver valuable information, which can help researchers gain an in-depth understanding of Se assimilation in plants in addition to its interaction with S.

For instance, the current review demonstrated that the studied brassica vegetables can accumulate high concentrations of Se, which is consequently assimilated to selenocompounds that have higher anticancer potential than S analogues. The findings are very promising and might lead to further experiments for the development of future application of nutraceutical substances and alternative plant-derived natural products with better chemopreventive properties.

Further investigations that focus on sulfate/selenate specificity in different brassica vegetables might help researchers gain a deeper understanding into the mechanisms of Se hyperaccumulation and assist the advancement in the field of anticarcinogenic agents and plant disease resistance. Hence, metals contamination has serious effect to human health and the ecosystem. Se enrichment in plants can be employed for the remediation of soils polluted with Se. In this regard, the advantage of Se hyperaccumulator plants to accommodate Se under high S levels could be transferred to other plants by following a molecular approach, which might result in the development of plant species with better applications in phytoremediation of Se-polluted soil and water.

Moreover, researchers have found that Se accumulates in plant cells including epidermal outgrowths of various kinds, known as trichomes, in addition to other epidermal cells, which can assist the better understanding of the hypothesis that Se hyperaccumulation has a role in plant defense mechanism. Trichomes exhibit an important role in plant defense mechanisms because of their different kinds of outgrowths, which play as significant structural barriers that provide a chemical line of defense against insects, pathogens, and herbivores [108].

The mechanism of action of some Se-biofortified brassica plants that exhibit chemopreventive potential, including studied broccoli in various maturity stages [80], is still unknown. Therefore, further experiments that could shed light and emphasize alert to Se biofortification as an alternative approach of chemoprevention are required.

Various analytical techniques have been developed to identify and quantify Se and its compounds in Brassicaceae. Among them, ICP showed a relatively high detection limit for Se, owing to its poor emission intensity in comparison with other elements. Additionally, the ICP technique can be coupled with mass spectrometry (ICP-MS) for both elemental and isotope ratio analysis. Moreover, HPLC-ICP-MS has been developed for Se speciation analysis [109,110].

GC-MS methods have been used to identify volatile Se metabolites, and headspace solid-phase microextraction (SPME) has been used to characterize volatile organic sulfur and Se compounds [111].

Chromatographic techniques such as HPLC combined with ICP-MS have been used to identify fractions containing Se, where authors determined the retention times and compared them with authentic reference compounds. Moreover, ESI in combination with ICP has been used to successfully identify Se metabolites. Nevertheless, the identification of selenocompounds in complex plant extracts is a significant challenge; in some studies, the minor Se species were not successfully identified and named as unknown. These metabolites could be responsible for the entire bioactivity including cancer inhibitory effects. Therefore, more sensitive analysis techniques are needed. Therefore, further investigations are required for the development of precise, exact, accurate, and green methods and analytical approaches for the elucidation of Se speciation in Brassicaceae to gain a deeper insight into Se/S assimilation in plants and humans.

## Figures and Tables

**Table 1:** Previous studies of Se and Se/S biofortification in brassica vegetables and their metabolites inducing chemopreventive inhibitory effects.

Studied Plant/Plants	Se or Se/S Application Methods	Observed Findings	References
*Arabidopsis thaliana* cv. Columbia and rapid cycling *B. oleracea*	Plants grown in hydroponic system. Four Na_2_SeO_4_ treatments (0.4, 0.8, 1.6, and 3.2 mg Se L^−1^) in addition to control treatment (no Se). One Se/S combination (0.8 mg Se L^−1^ and high S level 37 mg S L^−1^). All plants were harvested in 28−31 days	In both *B. oleracea* and *A. thaliana* the authors detected glucoraphanin, glucoiberin, glucoerucin, sinigrin, and indole-3-ylmethyl glucosinolates in response to all Se and higher sulfate doses. This might afford dietary Se and anticarcinogenic ITCs compounds at promising concentrations required to obtain chemopreventive properties	[79]
Broccoli (*B. oleracea* Italica)	Treatments of seeds and seedlings consisted of distilled water and 50 µM Na_2_SeO_4_, and inflorescences treated with distilled water and 1.5 mM Na_2_SeO_4_	The non-polar extracts of all samples exhibited antiproliferative effects. Se biofortified extract of broccoli seedlings with selenium showed cytocidal activity for a glioma cell line (U251, GI_50_ 28.5 mg L^−1^).	[80]
Salad rocket *(E. sativa* Mill.) and wild rocket *(Diplotaxis tenuifolia*)	Hydroponically grown plants under basal nutrient solution included MgSO_4_ at 1.5 µM in addition to other elements. At 30 days sodium selenate (Na_2_SeO_4_) was applied to the nutrient solution at 5, 10, 20 or 40 µM.	Se fertilization reduced the production of total GLS in *E. sativa*, consisted of aliphatic GLS, which synthesized from methionine. Additionally, higher amounts of phenolic compounds were decreased in *D. tenuifolia* in response to all Se levels, whereas in *E. sativa* the depletetion of phenolic compounds occurred only at higher Se concentrations.	[81]
Rapid-cycling *B. oleracea*	Plants grown in hydroponic system under different Se concentrations: 0, 1.0, 2.0, 3.0, 6.0, 7.2 or 9.0 mg L^−1^ Na_2_SeO_4_, S concentration was 246.48 mg L^−1^ MgSO_4_·7H_2_O.	The detection of 3-indolylmethyl glucosinolate and 2-propenyl glucosinolate in shoots under varied Se concentrations might offer chemopreventive effect.	[82]
*A. bisulcatus*, *S. pinnata* and *B. juncea*	Plants were cultivated for 8 or 6 months and irrigated 3 times a week with 40 μM SeO_4_^−2^. *B. juncea* were grown for one month and watered 3 times a week with 20 μM SeO_4_^−2^ for an extra month	Se was accumulated in the form of MeSeCys and γ-glutamyl-MeSeCys in the trichomes, which might show their capability of sequestration or biosynthesis of organo Se comounds in trichomes	[83]
Cabbage (*B. oleracea* var. *capitata* L. cv Jingfeng)	Sodium selenite (Na_2_SeO_3_) via foliage leaf spray (50, 100, or 150 μmol L^−1^) and MgSO_4_ via root irrigation (1 mM and 4 mM) and Na_2_SO_4_ treatments (3 mM), which were added with Hoagland’s nutrient solution for ten weeks.	GLS level was enhanced by low selenite (50 μmol L^−1^) and S (1 mmol L^−1^) concentrations or elevated selenite (100 μmol L^−1^) and S (4 mmol L^−1^) treatments.	[84]
cabbage (*B.oleracea* var. *capitata* L., cv. Pandion) and red cabbage (*B. oleracea* var. *capitata* L. f. rubra, cv. Erfurtsko rano)	Se was foliarly sprayed in the form of Na_2_SeO_4_ twice with 20 mg Se L^−1^ for cabbage and twice with 0.5 mg Se L^−1^ for red cabbage.	SeMet was the most abundant compound detected in all parts of cabbage and red cabbage; it constituted 94 and 55%, respectively, of the soluble Se concentration in the roots of both cabbages. In the stems and leaves of cabbage, SeMet was only 23% of the soluble Se content. In red cabbage SeMet was 80% and 41% of the soluble Se content in stems and leaves, respectively.	[87]
16 different brassica seeds cultivars including three broccoli cultivars, three cauliflower cultivars, three green cabbage cultivars, three Chinese cabbage cultivars, three kale cultivars and one Brussels sprouts	Seeds were soaked with 50 μM Na_2_SeO_4_, sprouts were treated with the same Se concentration every 24 h and harvested after 7 days	All sprouts treated with Se accumulate substantial amounts of MeSeCys. The lowest MeSeCys amounts were detected in sprouts of both green cabbage cultivars and brussels sprouts. Chinese cabbage had accommodated relatively high MeSeCys levels.	[52]
Broccoli sprouts *B. oleracea* var. italica	Seeds were grown in 10 ppm of Na_2_SeO_3_ solution to produce Se enriched sprouts or in deionized H_2_O to produce control broccoli sprouts	Selenium-enriched broccoli sprouts showed better cell proliferation inhibition than control broccoli sprouts including reducing prostate-specific antigen secretion and enhancing apoptosis of prostate cancer cells. Selenium-enriched sprouts but not normal sprouts, stimulate a downregulation of survival protein kinase B (Akt)/mammalian target of rapamycin (mTOR) pathway, which regulates various cellular processes	[92]
Broccoli (*B. oleracea* L. var. italica cv. Triathlon), cauliflower (*B. oleracea* L. var. botrytis cv. Liberty), and forage rape (*B. napus* cv. Maxima)	Plants were grown in soil and treated with 20 mL of 5.0 mM Na_2_SeO_4_ twice weekly for 4 weeks.	Three selenoglucosinolates were obtained including 3-(methylseleno)propylglucosinolate (glucoselenoiberverin), 4-(methylseleno) butylglucosinolate (glucoselenoerucin), and 5-(methylseleno)pentylglucosinolate (glucoselenoberteroin). Additionally, six organoselenium compounds were detected. Selenoglucosinolates levels and their aglycones including ITCs in forage rape, were up to 10% and 70%, respectively, of their S counterparts. Moreover, in broccoli, selenoglucosinolates concentrations and their aglycones including nitriles occurred up to 60% and 1300%, of their S counterparts.	[94]
Radish *Raphanus sativus*	Plants were grown under hydroponic conditions with addition of selenite or Se nanoparticles (1 mg L^−1^) for 40 days	More than 95% of the accommodated Se was incorporated into MeSeCys and SeMet	[96]
Kale *B. oleracea* A.	Plants were grown in cells contained Promix BX. Seven weeks after seeding, the cells were fertilized with Na_2_SeO_3_ or Na_2_SeO_4_ at a concentration of 10 µg g^−1^ Se for two weeks	MeSeCys was found to be the prominent Seleno compound found in kale and indicated to be responsible for its chemopreventive effect	[97]
White cabbage (*B. oleracea* L. var. capitata cv. Megaton)	Cabbages were shredded, and 0.5% NaCl and 0.3 mg of Na_2_SeO_3_/kg of fresh cabbage were added. Sauerkrauts without Se addition was prepared (control). Fermentation was performed by using indigenous microbiota of raw cabbage at room temperature (22–25 °C) for 7 days.	MeSeCys was found to be the major Se metabolite in Se-enriched sauerkraut	[99]
Black mustard (*B. nigra*)	Seeds cultivated in the seleniferous belt in the Punjab region (India) and the Se soil level is 2 to 7 mg kg^−1^	Selenoglucosinolates were identified as the main important group among Se compounds, which constituted 15% of the total existing Se and up to 50% of all present Se species. Other characterized Se metabolites were selenoamino acids, selenosugars, selenosinapine and selenourea derivatives	[100]
Kale *B. oleracea* var. sabellica L.	Seedlings were grown under hydroponic conditions with Hoagland’s solution and treated with 5, 10, 15, 30 and 45 μg mL^−1^ Na_2_SeO_3_ for 15 days and harvested every 5 days.	SeMet, and MeSeCys are found to be predominant seleno compunds	[101]
Broccoli *B. oleracea* L. var. Italica Group	Seeds soaked in Magenta boxes and Se treatments were 10, 25, 50, 75, and 100 μM as Na_2_SeO_4_ or Na_2_SeO_3_. S treatments were 0.1, 1, and 10 mM. Sprouts harvested in 7 days. In the subsequent two experiments 5 cultivars were used. One experiment with sprouts using the same procedure as above. The other one with mature broccoli plants in which seeds were planted in soil, additionally 100 mL of 1.5 mM Na_2_SeO_4_ applied to give a final concentration of 25 μM Se. 6 applications were performed 2× per week for 3 weeks	MeSeCys level in sprouts simultaneously enhanced with increasing Se treatments. Increasing S treatments diminished the total Se and MeSeCys levels in sprouts biofortified with selenate, but not in sprouts treated with selenite. Importantly, sprouts had a 6-fold higher glucoraphanin level than florets	[102]
Broccoli *B. oleracea* L.var. Marathon	Soil fertilization with increasing amounts of dried Se-enriched *S. pinnata* (~700 μg Se g^−1^ DW). Broccoli was then cultivated in triplicate as 14 day old transplants	Soluble Se metabolites in broccoli florets were 58% seleno-methionine, 15% selenocystine, 7.4% MeSeCys, 6% selenate and 3.1% selenite	[103]
Broccoli *B. oleracea* L.var. Marathon	Se-amended soil by using *S. pinnata* plant material as discriebed in Bañuelos et al. (2015) [103]	The identified Se species constituted an average of 50, 11, and 5% as SeMet, SeCys, and MeSeCys, respectively, in addition to unknown compounds	[104]
Indian mustard (*B. juncea*)	Plants grown in hydroponic conditions and after 30 days Na_2_SeO_4_ or Na_2_SeO_3_ were used to obtain a concentration of 5 µg Se mL^−1^ and the S concentration was 2 mM MgSO_4_	The identified Se species were MeSeCys (3.1%) in shoot biofortified with selenate, (10.7%) and S-(methylseleno)cysteine was found in both shoot and root supplemented with selenate or selenite. The percentage of SeMet was 27.8 and 43.1% in both selenate and selenite treated roots, respectively. Additionally, SeMet found in shoot biofortified with selenate and selenite were 10.7% and 34.2%, respectively.	[105]

## Abbreviations

SeMet: selenomethionine; SeCys: selenocysteine; CH_3_SeH: methylselenol; MeSeCys: Se-methylselenocysteine; DDSs: redox-responsive drug delivery systems; Na_2_SeO_3_: Sodium selenite; Na_2_SeO_4_: sodium selenate; γ-glutamyl-MeSeCys: γ-glutamyl-Se-methylselenocysteine; GLS: glucosinolates; ITCs: isothiocyanates; DMBA: 7,12-dimethylbenz(a)anthracene; ROS: reactive oxygen species; GSH: glutathione; GS–SeH: glutathione selenopersulfide; IC_50_:The half maximal inhibitory concentration; GS–Se–SG: selenodiglutathione; DW: Dry weight; CS–SeNPs: chitosan-modified Se nanoparticles; AFFFF: asymmetrical flow field flow fractionation; ESI: electrospray ionization; APCI: atmospheric pressure chemical ionization; HPLC-ICP-MS: high-performance liquid chromatography inductively coupled plasma-mass spectrometry; TEM: transmission electron microscopy; SPME: solid-phase microextraction.
